# Self−Assembling Nanovaccine Fused with Flagellin Enhances Protective Effect against Foot−and−Mouth Disease Virus

**DOI:** 10.3390/vaccines11111675

**Published:** 2023-11-02

**Authors:** Chenchen Pei, Hu Dong, Zhidong Teng, Sumin Wei, Yun Zhang, Shuanghui Yin, Jianli Tang, Shiqi Sun, Huichen Guo

**Affiliations:** 1 State Key Laboratory for Animal Disease Control and Prevention, College of Veterinary Medicine, Lanzhou University, Lanzhou Veterinary Research Institute, Chinese Academy of Agricultural Sciences, Lanzhou 730046, China; 2 Gansu Province Research Center for Basic Disciplines of Pathogen Biology, Lanzhou 730046, China; 3 College of Animal Science, Yangtze University, Jingzhou 434023, China

**Keywords:** foot−and−mouth disease, flagellin, self−assembling, nanoparticles, nanovaccine

## Abstract

Nanovaccines based on self−assembling nanoparticles (NPs) can show conformational epitopes of antigens and they have high immunogenicity. In addition, flagellin, as a biological immune enhancer, can be fused with an antigen to considerably enhance the immune effect of antigens. In improving the immunogenicity and stability of a foot−and−mouth disease virus (FMDV) antigen, novel FMDV NP antigens were prepared by covalently coupling the VP1 protein and truncated flagellin containing only N−terminus D0 and D1 (N−terminal aa 1–99, nFLiC) with self−assembling NPs (i301). The results showed that the fusion proteins VP1−i301 and VP1−i301−nFLiC can assemble into NPs with high thermal tolerance and stability, obtain high cell uptake efficiency, and upregulate marker molecules and immune−stimulating cytokines in vitro. In addition, compared with monomeric VP1 antigen, high−level cytokines were stimulated with VP1−i301 and VP1−i301−nFLiC nanovaccines in guinea pigs, to provide clinical protection against viral infection comparable to an inactivated vaccine. This study provides new insight for the development of a novel FMD vaccine.

## 1. Introduction

Foot−and−mouth disease (FMD) is an acute infectious disease, which primarily infects cloven−hoofed animals such as pigs, cattle, and sheep. FMD is caused by the foot−and−mouth disease virus (FMDV) [1,2,3,4,5]. FMD occurs worldwide, seriously affects the livestock industries, and causes significant economic losses [6,7]. Traditional vaccines are effective against FMD, in combination with other control measures, promoting many countries and regions to achieve an epidemic−free standard [8]. Compared with the traditional inactivated vaccines, new subunit vaccines have a simple composition, good safety, and convenient clinical application [9,10]. Given the relatively weak immunogenicity, the development of an FMD subunit vaccine is relatively lagging behind. At present, nanotechnology has been paid attention to in the field of new vaccine development. Nanotechnology has a large load, and stable structure, and it can play a role in immune enhancement, which is considered as an effective strategy to improve the immunogenicity of epitope−based vaccines [11,12].

Nanoparticles (NPs) are non−infectious, easy in synthesis, and uniform in size; thus, they are widely used in biomedical imaging [13,14,15], targeted drug delivery [16,17,18] and vaccine preparation [19,20]. At present, the self−assembly of viral capsid proteins into NPs has been proposed to produce virus−like −particles (VLPs), such as human papillomavirus [21] and hepatitis B virus [22], which have been successfully applied in clinical practice. Previous reports have shown that FMD VLPs have been successfully developed and have produced good immune protection [23]. Given the lack of non−structural proteins compared with whole viruses, VLPs can be used to differentiate infected from vaccinated individuals (DIVA) to distinguish between immunized and naturally infected animals [24], which is considered as a potential alternative to inactivated vaccines [25]. However, the specific immune response induced by VLPs are weaker than inactivated vaccines, and the thermal stability of VLPs are poorer than that of native proteins. Biomineralization using metallics, not only can improve thermal stability, but also can effectively activate antigen−presenting cells (APCs) [26,27]. Although biomineralization significantly improves the thermal stability and immune response of a VLP, it has certain limitations in biocompatibility. Therefore, finding more comprehensive design strategies is necessary.

To date, many bio−derived protein self−assembling NPs (SAPNs) with viral structures have been designed through computer−software calculations and simulations, which have received considerable attention in the research and development of nanovaccines because of their good histocompatibility and easy modification. For example i301, a self−assembling protein designed on the basis of the 2−ketone−3−deoxy−6−phosphoglucolic−acid (KDPG) aldolase from the hyperthermophilic archaea, forms a high−order hyperstable dodecahedron nanocage with 60−mer through five mutations at the interface of the wild−type protein trimer [28]. The icosahedron has a robust genetic−fusion ability, with its N−terminus and C−terminus being able to connect proteins, and display them outside and inside the nanocages and maintain functions, which should be quite practical in vaccine design and synthetic biology.

Nanovaccines couple antigens with NPs through chemical modification and gene fusion. With NPs as the carriers, multiple copies of the target antigen can be displayed on its surface with correct conformation [11], and cross presentation can be promoted through major histocompatibility complex (MHC) I and MHC II pathways, thereby promoting the response of B lymphocytes and T lymphocytes, and ensuring that the nanovaccines have good immunogenicity [29,30,31]. Moreover, antigen−recognition uptake by cells is the initial step to produce robust adaptive immunity; NPs have significant advantages because of their small particle size, and their higher antigen−uptake efficiency indicates better specific immune response [32].

In general, biological immune enhancers, such as lipopolysaccharide [33], liposome [34], cytokines [35] and flagellin [36], can improve the immune effect of vaccine. Notably, flagellin, which has four domains, D0, D1, D2, and D3, arranged in a sequence to form a boomerang−like structure [37], can specifically bind to the toll−like receptor 5 (TLR5) of the host as pathogen−associated molecular patterns (PAMPs), and can mediate the expression of factors including Interleukin−1 receptor−associated kinase−4, TGF−β activated kinase−1 (TAK1) and nuclear factor kappa−B (NF−κB) by binding to the key junction molecule myeloid differentiation factor 88 (MyD88) [38,39,40], leading to the activation of immune cells [41]. Then, the activated immune cells secrete and express a variety of cytokines and costimulatory factors such as interleukin 6 (IL−6), interleukin 12 (IL−12), tumor necrosis factor α (TNF−α), and interferon γ (IFN−γ) [42]. In addition, a comparative analysis of the structure and functional studies of different bacterial flagellins showed that D0 and D1 are highly conserved, whereas D2 and D3 showed greater differences [43]. Research has shown that the truncate of flagellin containing only N−terminus D0 and D1 (N−terminal aa 1–99, nFLiC) can preserve the full ability to activate TLR5 because flagellin primarily relies on a region of 13 residues in D1 to bind to TLR5 [44,45]. However, the absence of D2 and D3 does not affect the recognition of TLR5 [46]. This phenomenon not only reduces the adverse reactions caused by intact flagellin, but also greatly increases the flexibility of flagellin design and fusion because of the decrease in molecular weight.

As the main structural protein of FMDV, VP1 can induce cellular and humoral immunity, producing protective neutralizing antibodies, and VP1 is a key component determining the antigenicity of the virus. The design of nanovaccines using VP1 and self−assembled proteins not only ensures immunogenicity and stability, but also improves the biocompatibility of NPs.

In the present study, two NPs (VP1−i301 and VP1−i301−nFLiC) were prepared by covalently coupling the FMDV VP1 protein and nFLiC with the i301 scaffold protein using the prokaryotic expression system. The self−assembly efficiency, thermal stability and the adjuvant effect on APCs in vitro of NPs were characterized subsequently. Furthermore, the protection and immunogenicity of NPs against FMDV challenge were evaluated in guinea pigs. The results showed that NPs have better thermal stability and cellular uptake ability in vitro compared with monomeric VP1. In addition, NPs can effectively induce immune response in vivo and protect guinea pigs from FMDV challenge compared to inactivated FMDV, significantly outperforming monomeric VP1.

## 2. Materials and Methods

### 2.1. Construction of Recombinant Positive Plasmids

i301 (PDB: 5KP9) and truncated nFLiC (1–99 aa) of flagellin (PDB ID: 1UCU) were obtained from a protein data bank (https://www.rcsb.org/) (accessed on 16 September 2020), and VP1 was obtained from an FMDV−type−O strain O/BY/CHA/2010 (GenBank accession No. JN998085.1). The FMDV−type−O strain O/BY/CHA/2010 was classified in a Mya98 lineage, SEA topotype [47]. Genes were codon optimized and synthesized into pSMA [23] to generate pSMA−VP1, pSMA−VP1−i301, and pSMA−VP1−i301−nFLiC by Jiangsu Genscript Biotechnology Co., Ltd. (Nanjing, China). To avoid steric hindrance and allow for appropriate folding, a small ubiquitin−like modifier (SUMO) tag was introduced between His and VP1, and flexible linkers (GGS) were introduced between target proteins i301 and nFLiC.

### 2.2. Expression and Purification of Recombinant Proteins

The plasmids were transformed into *Escherichia coli* BL21(DE3), which were cultured in an LB medium containing 50 μg/mL of ampicillin and induced with 0.5 mM Isopropyl−β−D−thiogalactoside at 20 °C for 16 h for protein expression. Cells were collected and centrifuged at 5000× *g* for 30 min at 4 °C. Pellets were resuspended in lysis buffer (50 mM Tris−HCl, 300 mM NaCl, pH 8.0). The lysate was sonicated on ice for 15 min with rounds of 3 s on and 3 s off, and then centrifuged at 12,000× *g* for 30 min at 4 °C. The supernatant of recombinant protein obtained using centrifugation was loaded into Ni NTA columns, washed with washing solution containing different concentrations of imidazole (5 mM, 20 mM, and 50 mM), and finally target proteins were eluted with 500 mM imidazole buffer. Afterwards, SUMO protease was used to cleave SUMO tags to release target fusion proteins. The purified proteins were analyzed with sodium dodecyl sulfate polyacrylamide gel electrophoresis (SDS−PAGE) and Western blot. The FMDV VP1 rabbit anti−monoclonal antibody (1:2000) was incubated with a PVDF membrane at 37 °C for 2 h, followed by TBST rinsing to remove unbound antibodies. HRP−labeled goat−anti−rabbit IgG (diluted 1:2000, Sigma−Aldrich Corporation, St. Louis, MO, USA) was added and incubated at 37 °C for 1 h. TBST rinsing was performed three times, and enhanced chemiluminescence (ECL) staining was used to observe the results.

### 2.3. Physicochemical Conditions and Properties of NPs

Purified VP1−i301 and VP1−i301−nFLiC fusion proteins were filtered using a 0.22 μM syringe filter, subsequently dialyzed against assembly buffer (500 mM NaCl, 40 mM Tris, 5% lycerin, pH 8.0), and placed on magnetic stirrers at 300 rpm and 4 °C overnight. The size and characteristics of the NP self−assembly were observed using dynamic light scattering (DLS, ZEN3600, Malvern, PA, USA) and transmission electron microscopy (TEM, HT7700, Hitachi Corporation, Tokyo, Japan).

### 2.4. Thermal Stability Detection of NPs

To evaluate thermal stability under different conditions, stocks of VP1−i301, VP1−i301−nFLiC NPs and VP1 monomer proteins were incubated at 25 °C, 35 °C, 45 °C, 55 °C, 65 °C or 75 °C for 1 h (The initial concentration of each protein sample is 500 μg/mL). Subsequently, the protein samples were centrifuged at 15,000× *g* for 10 min at 25 °C, and the hydrated particle−size change of NPs was detected using DLS. The supernatant was taken for quantitative analysis of soluble protein samples; soluble fractions at 4 °C were defined as 100% [48]. As for long−term storage−stability analysis, stocks of VP1−i301 NPs, VP1−i301−nFLiC NPs, and VP1 monomer proteins were aseptically filtered and incubated at 37 °C using a thermal cycler. At the indicated time points, the sample was centrifuged to remove aggregates and the effective antigen was quantitatively analyzed using dot blotting. In particular, a small square NC membrane was taken and flattened on a clean desktop; several square grids with a size of 1 cm^2^ were drawn; and 5 µL of samples were pointed at the positions shown in the grids. Then, the samples were left to stand for 10–15 min, so the samples could be completely attached to the membrane. Afterward, 5% skimmed milk was used as a seal on the shaking table at room temperature for 1 h, and the membrane was incubated with the FMDV VP1 rabbit anti−monoclonal antibody for 2 h at 37 °C, and rinsed with TBST to remove the unbound antibody. Next, the samples were added with HRP−labeled goat−anti−rabbit IgG (diluted 1:2000, Sigma, USA), incubated at 37 °C for 1 h, and washed with TBST three times. The ECL method was used to observe the results.

### 2.5. Cellular Uptake Assay

In evaluating the uptake efficiency of NPs in cells, BHK−21 and RAW264.7 cell suspensions of the logarithmic growth phase were seeded into a 12−well plate (1 × 10^6^ cells/well) and incubated at 37 °C with 5% CO_2_ until cell density reached 80%. Then, the cells were stimulated with PBS, VP1−i301 NPs (10 μg), VP1−i301−nFLiC NPs (10 μg), or monomeric VP1 (10 μg) and incubated for 1, 2, 4 and 6 h. Cells were washed three times with ice−cold PBS (Solarbio, Beijing, China) to remove unbound residual proteins before collecting for the following process. Then, the uptake of NPs by cells was measured using Western blot. Rabbit−serum−containing antibody specific to FMDV VP1 was used as the primary antibody (1:2000), followed by incubating with HRP−labeled goat−anti−rabbit IgG (1:2000).

### 2.6. Separation and Generation of Bone−Marrow−Derived Cells (BMDCs)

BMDCs were obtained from the femoral bone marrow of C57BL/6 mice with RPMI−1640 medium (Gibco, Grand Island, NY, USA) and centrifuged at 1200 rpm for washing. Red blood cells were lysed in Red Blood Cell Lysis Buffer (Solarbio, Beijing, China), and the remaining cells were collected. Subsequently, the cells were resuspended in 12−well plates with RPMI−1640 complete medium containing 10% FBS (Gibco, Penrose, Auckland, New Zealand), 20 ng/mL of IL−4 (Minneapolis, USA), 30 ng/mL of GM−CSF, and 50 μM β−mercaptoethanol(Sigma−Aldrich Corporation, St. Louis, MO, USA)—−each substance was added into wells separately. After culturing for 7 days, most cells obtained a typical dendritic morphology as described previously, and such cells can be used for subsequent experiments [27].

### 2.7. TLR5 Pathway Activation Assay

PBS, VP1−i301 NPs (10 μg), VP1−i301−nFLiC NPs (10 μg) and monomeric VP1(10 μg) were separately added to stimulate BMDCs cultured in 12−well plates. Based on the previously described method [26], the expression level of downstream molecules of TLR5, including MyD88, TAK1, and NF−κB (CST, 3 Cell Signaling Technology, Inc. Danvers, MA, USA), was measured using Western blot.

### 2.8. Expression of Marker Genes and Cytokines by BMDCs

The effect of NPs on the expression level of surface markers and cytokines of BMDCs was investigated. BMDCs were stimulated with VP1−i301 NPs (10 μg), VP1−i301−nFLiC NPs (10 μg) or monomeric VP1 (10 μg), and total RNA was extracted. cDNA was synthesized with PrimeScript reverse transcriptase (Takara, Dalian, China) in accordance with the user manual. Quantitative real−time PCR (qPCR) was performed using TB Green Premix Ex Taq (Takara, Dalian, China) as described by the manufacturer’s manual. The primer sequences for qPCR are shown in Table 1. β−actin was used as an internal reference to normalize mRNA expression, and relative expression data were analyzed using the 2^−ΔΔCt^ method.

### 2.9. Animal Immunization and Challenge Trial

A total of 30 male Hartley guinea pigs (body weight of 200–300 g) were randomly divided into five groups with six animals per group. Each group was immunized intramuscularly with PBS, VP1 (25 μg), VP1−i301 NPs (25 μg), VP1−i301−nFLiC NPs (25 μg), and inactivated FMDV (10 μg), and each vaccine was emulsified with ISA−206 adjuvant. In addition, sera samples were collected at 7, 14, 21, and 28 days post−immunization (dpi). According to a previous report, specific antibody levels were detected with sandwich ELISA [49], and the neutralizing antibody titer was analyzed using a serum neutralization test (SNT) [27]. Lymphocyte proliferation was detected using the CellTiter 96 Aqueous Non−Radioactive Cell Proliferation Assay (Promega, Beijing, China) [26]. Cytokine levels of TNF−α, IFN−γ, and IL−12 in the blood of guinea pigs were quantified with qPCR, and the primer sequences are shown in Table 2. To explore the protective rate of NP vaccines, at 28 dpi, guinea pigs were challenged with 200 µL of FMDV−type−O strain O/BY/CHA/2010 (GenBank accession No. JN998085.1) at a 100−guinea−pig intraperitoneal 50% (100 GPID50) infectious dose via inoculation of the left heel pads. Afterward, the guinea pigs were continuously observed for 7 days [23].

### 2.10. T lymphocyte Proliferation Response

Guinea−pig spleen lymphocytes were isolated and stimulated with concanavalin, and cultured in a 37 °C incubator for 72 h. Afterwards, PBS, VP1−i301 NPs (10 μg), VP1−i301−nFLiC NPs (10 μg), and monomeric VP1 (10 μg) were separately added to stimulate lymphocytes cultured in 12−well plates. The lymphocyte proliferation reaction was detected using a CellTiter 96 Aqueous Non−Radioactive Cell Proliferation Assay (Promega, Beijing, China), and the absorbance was measured at a 490 nm wavelength of the microplate reader.

### 2.11. Statistical Analysis

Values were expressed as mean ± SD (standard error of the mean), and data analyses was performed using Student’s *t* test when two groups were compared, or with one−way analysis of variance for more than two groups. Origin Pro 8.5 was used for chart drawing. SPSS 24 was used for comparative analysis. *p* < 0.05 was considered a significant difference.

## 3. Results

### 3.1. Expression and Characterization of NPs

**Figure 1:**
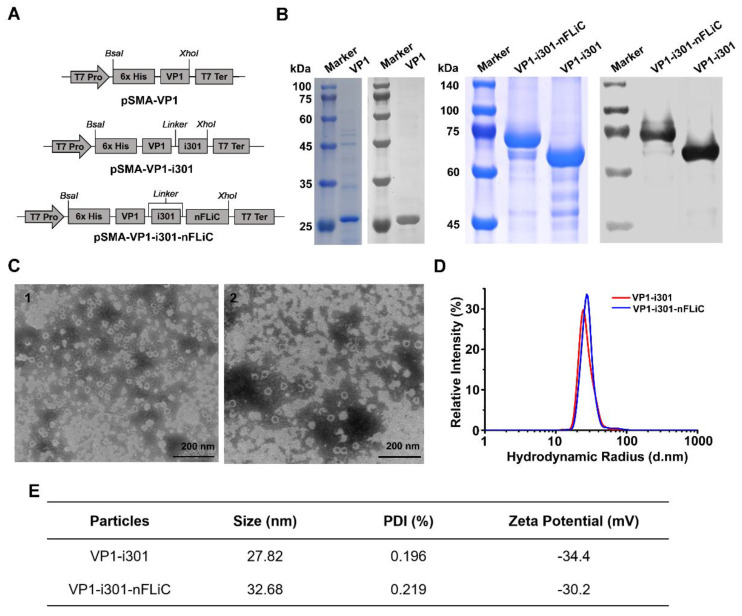
Expression and self−assembly of NPs. (**A**) Schematic diagram of VP1, VP1−i301 and VP1−i301−nFLiC expression cassettes cloned in the pSMA expression vector. (**B**) SDS−PAGE and Western blot analyses of purified fusion proteins. (**C**) TEM image of VP1−i301 (1) and VP1−i301−nFLiC NPs (2). (**D**) Hydrodynamic diameter and size distribution of VP1−i301 and VP1−i301−nFLiC NPs were analyzed with DLS. (**E**) The Size, PDI, and Zeta Potential data of VP1−i301 and VP1−i301−nFLiC NPs.

To investigate the self−assembly and immunogenicity of VP1−i301 and VP1−i301−nFLiC fusion proteins, two plasmids were constructed using the target protein VP1 of FMDV−type−O strain O/BY/CHA/2010, N−terminal truncate of flagellin (nFLiC), and self−assembly protein i301 (Figure 1A). The fusion proteins were successfully expressed in *Escherichia coli* BL21 (DE3) cells and subjected to purification using 6× His tag mediated−metal affinity chromatography. The target−protein bands were consistent with the predicted sizes analyzed using SDS−PAGE (Figure 1B), which are observed at 25 kDa (VP1), 63.3 kDa (VP1−i301), and 74.3 kDa (VP1−i301−nFLiC), and the target proteins were confirmed to react significantly with the monoclonal antibody of VP1 with Western blot (Figure 1B). The NPs were assembled after dialyzing against the assembly buffer at 4 °C for 24 h and were examined using TEM. The results showed that the fusion proteins VP1−i301 and VP1−i301−nFLiC could self−assemble into uniform NPs with a particle size of 25–30 nm (Figure 1C). Notably, the hydrodynamic size of the dissolved samples measured with DLS is different from that measured using TEM analysis in a dried state, and so the hydrated particle size of most NPs is slightly larger than that measured with TEM analysis. The results showed that the hydrated particle sizes of VP1−i301 and VP1−i301−nFLiC NPs were approximately 27.82 and 32.68 nm, respectively (Figure 1D), and were consistent with expectations. Poly dispersity indexes (PDI) of VP1−i301 and VP1−i301−nFLiC NPs were 0.196% and 0.219%. Zeta potentials of VP1−i301 and VP1−i301−nFLiC NPs were −34.4 mV and −30.2 mV (Figure 1E).

### 3.2. Thermal Stability Analysis of NPs

**Figure 2:**
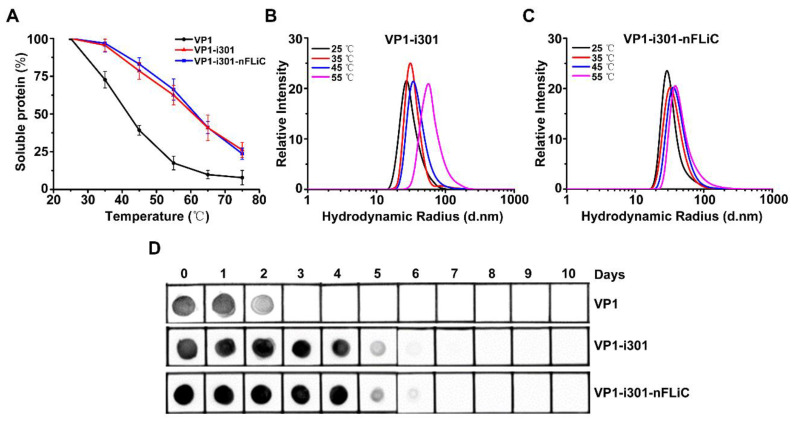
Thermal stability analysis of NPs. (**A**) NPs remained soluble at high temperatures. (**B**) Hydrodynamic diameter and size distribution of VP1−i301 NPs at different temperatures were analyzed with DLS. (**C**) Hydrodynamic diameter and size distribution of VP1−i301−nFLiC NPs at different temperatures were analyzed using DLS. (**D**) Dot blot analysis of the stability of monomeric VP1, VP1−i301, and VP1−i301−nFLiC NPs stored for 10 days at 37 °C.

At present, cold−chain transportation is a major challenge for the cost and efficacy of vaccines in developing countries. To verify the thermal stability, VP1−i301 NPs, VP1−i301−nFLiC NPs, and monomeric VP1 were incubated at different temperatures for 1 h, and the proportion of proteins in the soluble fraction was measured. The solubility of two NP groups was significantly higher than monomeric VP1 at different temperatures (Figure 2A). The solubility of both NP groups was about 65% at 55 °C, whereas that of VP1 was only about 20%. Apart from solubility, we also explored whether the remaining NPs could maintain a narrow size distribution. When the temperature increased, the hydrodynamic radius increased, indicating good tolerance (Figure 2B,C). In simulating and verifying the stability of storage, the samples were placed at 37 °C for a long time and received regularly, and effective antigen content was analyzed using Dot blotting. We found that the degradation rate of NP groups was significantly slower than monomeric VP1, with a significant thermal stability (Figure 2D).

### 3.3. Cellular Uptake with NPs

**Figure 3:**
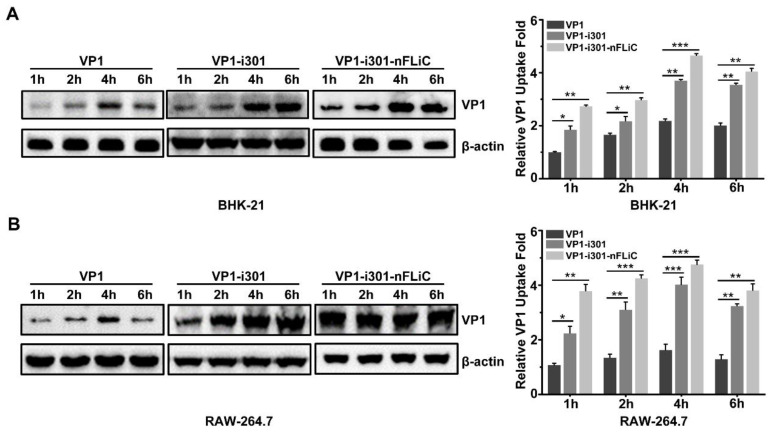
Cellular uptake of NPs. Efficiency of the cellular uptake of NPs and control VP1 protein. The BHK−21 (**A**) and RAW−264.7 (**B**) cells were stimulated with different protein samples and analyzed for the presence of internal VP1 with Western blot at 1, 2, 4, and 6 h. The original Western blots and relative VP1 levels determined using densitometry are shown. β−actin was used as a loading control for normalization. Experiments were performed in triplicate and data are presented as mean ± SD. * *p* < 0.05; ** *p* < 0.01, *** *p* < 0.001.

To determine the uptake efficiency of NPs, BHK−21 and RAW264.7 cells were used for experiments, and antigen uptake was detected with Western blot (Figure 3A,B). The results showed that both NPs could be effectively taken up by both cell types, and the amount of NPs taken up by the cells gradually increased with incubation time. NP groups could easily enter the cells, and uptake was significantly higher than the monomeric VP1, which indicated that the formation of NPs promotes the entry of antigens into cells.

### 3.4. NPs Induce TLR5−Mediated Inflammation in BMDCs

**Figure 4:**
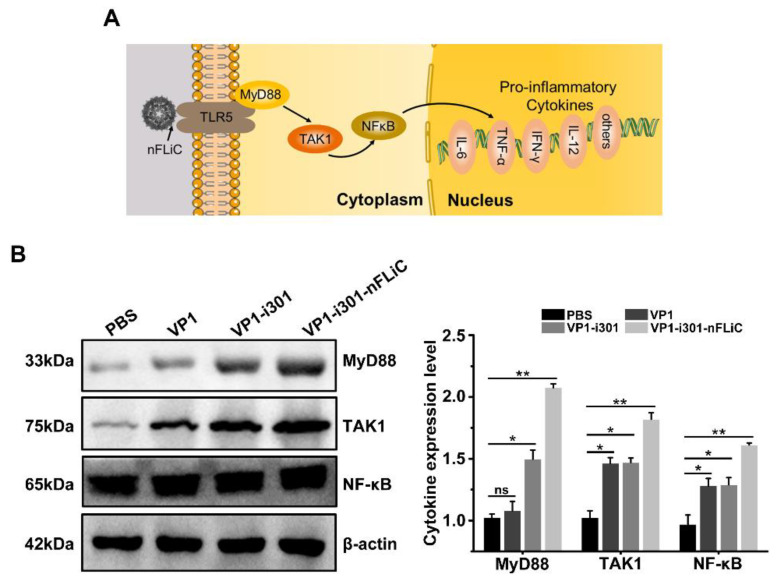
Expression of TLR5 pathway proteins. (**A**) Schematic diagram of TLR5 pathway molecules. (**B**) Immature BMDCs were stimulated with PBS, VP1, VP1−i301−nFLiC NPs, or VP1−i301−nFLiC NPs for 8 h. The expression of the TLR5 pathway molecules in BMDCs was detected using Western blot. β−actin was used as a loading control for normalization. Experiments were performed in triplicate and data are presented as mean ± SD. ns, *p* > 0.05, * *p* < 0.05; ** *p* < 0.01.

TLR5 can be widely expressed in a variety of immune cells, including DCs, T cells, monocytes, and macrophages. In addition, the schematic diagram of TLR5 pathway molecules is shown in Figure 4A. To confirm the activation of the TLR5 downstream signal in BMDCs mediated with VP1−i301−nFLiC NPs, the expression level of related molecules in the MyD88 pathway was determined using Western blot. The results showed that the expression level of proteins in the MyD88 pathway (MyD88, TAK1, and NF−κB) was significantly enhanced after BMDCs were stimulated with VP1−i301−nFLiC NPs compared with VP1−i301 NPs and monomeric VP1 (*p* < 0.05, Figure 4B).

### 3.5. NPs Stimulated Cytokine Production in BMDCs

**Figure 5:**
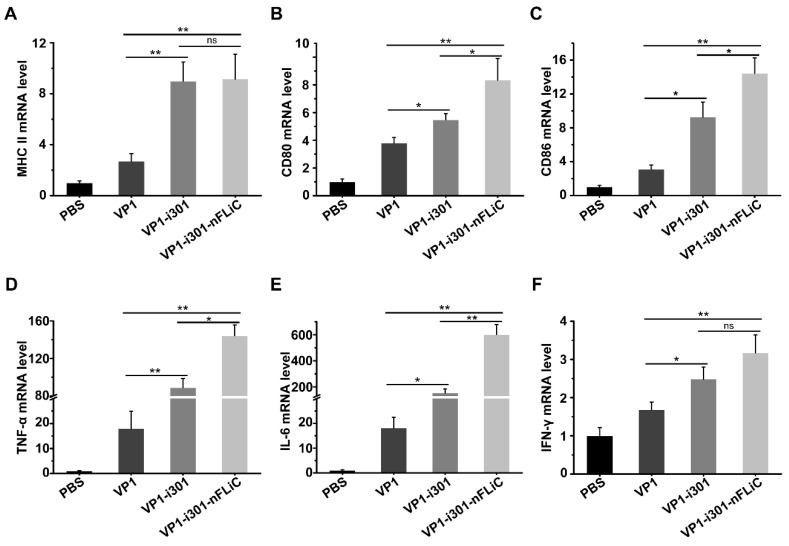
Detection of surface markers and cytokine expression of BMDCs. Total RNAs were extracted from VP1−, VP1−i301 NP−, or VP1−i301−nFLiC NP−stimulated BMDCs. mRNA levels of surface marker MHC II (**A**); costimulatory molecules CD80 (**B**) and CD86 (**C**); and pro−inflammatory cytokines TNF−α (**D**), IL−6 (**E**), and IFN−γ (**F**) were determined using q−PCR. β−actin was used as a loading control for normalization. Experiments were performed in triplicate and data are presented as mean ± SD. ns, *p* > 0.05; *, *p* < 0.05; **, *p* < 0.01.

To explore the effect of NPs on BMDCs, we tested the transcription of surface markers and cytokines expressed by BMDCs stimulated with VP1, VP1−i301 NPs, or VP1−i301−nFLiC NPs. As shown in Figure 5A–C, the expression level of the surface marker MHC II and costimulatory molecules CD80 and CD86 of BMDCs stimulated with VP1−i301 NPs or VP1−i301−nFLiC NPs was significantly higher than that of the VP1 group, and the upregulation of these genes indicated the maturation of BMDCs. In addition, we found that the proinflammatory cytokines in the VP1−i301 and VP1−i301−nFLiC NP groups were significantly higher than those in the VP1 group (*p* < 0.05). Moreover, TNF−α and IL−6 induced with VP1−i301−nFLiC NPs were increased by about 10−fold and 30−fold, respectively, compared with the monomeric VP1 in MBDCs (Figure 5D,E). Therefore, many cytokines released by activated BMDCs were directly or indirectly involved in immune responses, and such cytokines can play a critical role in enhancing the immunogenicity of antigens. NPs and nFLiC proteins can promote the maturation of BMDCs and upregulate the expression level of related factors, which can enhance the immune response.

### 3.6. Enhanced Immune Response Induced by Nanovaccines in Guinea Pigs

**Figure 6:**
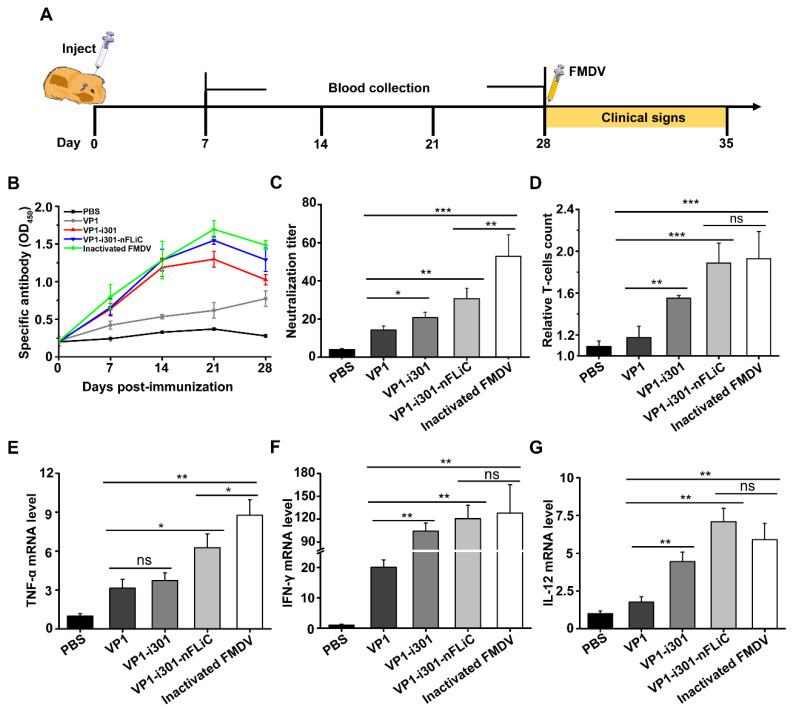
Animal experiment and effect evaluation. Blood samples were collected at 7, 14, 21, and 28 dpi (days post−immunization) for detecting the specific antibodies and neutralizing antibodies in guinea pigs. Spleen lymphocytes were isolated to detect the proliferation of T lymphocytes. (**A**) The procedure of animal experimental immunization. (**B**) Detection of specific antibodies. (**C**) Detection of neutralizing antibodies. (**D**) Detection of T lymphocyte proliferation. (**E**–**G**) Total RNAs of guinea pigs were isolated at 28 dpi from the blood, and mRNA levels of TNF−α (**E**), IFN−γ (**F**), and IL−12 (**G**) were determined using q−PCR. GAPDH was used as a loading control for normalization. Experiments were performed in triplicate, and data are presented as mean ± SD. ns, *p* > 0.05, *, *p* < 0.05; **, *p* < 0.01; ***, *p* < 0.001.

The specific immune response induced by NPs was further evaluated in guinea pigs. The specific immunization procedures are shown in Figure 6A. The specific antibodies and neutralizing antibodies against FMDV were detected using sandwich ELISA and SNT. The result of ELISA showed that specific antibody levels of VP1−i301 NPs, VP1−i301−nFLiC NPs, and inactivated FMDV−vaccinated groups increased over time and reached the highest level at 21 dpi, and the level of inactivated FMDV was higher than that of the VP1−i301−nFLiC NP and VP1−i301 NP groups (*p* > 0.05). The VP1 specific antibodies in both NPs and inactivated FMDV groups were significantly higher than that of the VP1 group (*p* < 0.05, Figure 6B). In addition, the results of neutralizing antibodies were similar to those of specific antibodies. The inactivated FMDV group produced the highest number of neutralizing antibodies which was significantly higher than others. Both NP groups stimulated significantly higher level of neutralizing antibodies than the VP1 group (Figure 6C). Then, spleen lymphocytes were isolated, and the effect of NPs on the proliferation of T lymphocytes was evaluated. As shown in Figure 6D, the relative T−cell count of the inactivated FMDV and VP1−i301−nFLiC NP groups was the highest (*p* > 0.05), followed by that of the VP1−i301 NP group (*p* < 0.05), and the VP1 group elicited the lowest neutralizing−antibody level (*p* < 0.01).

We further determined the effect of NPs on cellular immune response and inflammatory response by detecting cytokines. The secretory inflammatory factors which play a key role in regulating cell−mediated immunity, such as TNF−α, which is a core indicator of the activation of APCs, was upregulated significantly in NPs and FMDV−inactivated groups (Figure 6E). Compared with the VP1 group, NPs and inactivated−FMDV groups significantly enhanced IFN−γ and IL−12 cytokines (*p* < 0.01; Figure 6F,G), which are primarily responsible for the proliferation and differentiation of T cells and the production of cytokines in vivo. Based on these results, i301 NP and nFLiC can induce high levels of TNF−α, IFN−γ, and IL−12, which may promote immune responses.

### 3.7. Protective Efficacy against FMDV Challenge Stimulated by Nanovaccines

All the features of the nanovaccines described above contribute to in vivo protection against FMDV in guinea pigs. The guinea pigs were challenged with FMDV virus with a dose of 100 GPID_50_ at 28 dpi and continuously observed for one week. As shown in Table 3, the protection rates of the inactivated−FMDV and VP1−i301−nFLiC NP groups were 83.3%, and the VP1−i301 NPs group was 66.7%, which were higher than that of VP1 group (33.3%). The results showed that both nFLiC and i301 NPs could enhance the immune protection of guinea pigs.

All the above−mentioned features of the nanovaccines contribute to in vivo protection against FMDV in guinea pigs. The guinea pigs were challenged with FMDV−type−O strain O/BY/CHA/2010 (GenBank accession No. JN998085.1) at a dose of 100 GPID_50_ at 28 dpi and continuously observed for one week. As shown in Table 3, the protection rates of inactivated FMDV and the VP1−i301−nFLiC NP groups were 83.3%, and that of the VP1−i301 NP group was 66.7%, which were higher than that of the VP1 group (33.3%). The results showed that nFLiC and i301 NPs could enhance the immune protection of guinea pigs.

## 4. Discussion

Subunit vaccines have simpler antigen components and higher safety than traditional whole−pathogen−based antigens and genetic vaccines. Most DIVA vaccines were designed with these advantages to distinguish between a wild−type viral infection and artificial immunity [50,51,52,53]. However, relevant reports have shown that the homologous subunit vaccines are facing various challenges in efficiency [54,55,56], which includes that they are effective in eliciting predominant humoral immunity, but they fail to induce sufficient cellular immunity to remove intracellular pathogens [57,58]. Therefore, developing an efficient and safe universal−DIVA−vaccine design platform is necessary. Increasing reports have shown that SAPNs, as an efficient delivery system, have greatly promoted the development of novel vaccines [59,60,61]. SAPNs have significant advantages in particle uniformity, modifiability, histocompatibility, and biodegradability, and such NPs can be used as ideal scaffolds for the targeted display of heterologous antigens to generate chimeric nanovaccines against multiple pathogens [62,63]. The antigen can be displayed in high copy on the surface, which greatly enhances the stability and immunogenicity of the vaccines. However, given the small size of most NPs, large inserts (antigen/epitope size) may destroy the stability of NPs and even lead to their inability to assemble, which to a certain extent hinders the widespread use of NPs−based vaccine design [64].

In this study, a vaccine strategy based on the i301 self−assembled protein is proposed, and FMDV VP1 is genetically fused to the N−terminal of i301 (VP1−i301). As shown using TEM and DLS, the fusion protein can self−assemble into uniform NPs in vitro, consistent with previously reports [65,66]. The FMDV VP1 structural protein contains very important neutralizing antigenic epitopes. One is the RGD motif (AA: 140–160), which is a key recognition site for virus entry into cells. The other is a short peptide located at the C−amino terminus of VP1 (aa: 200–213), which can also induce protective immune responses [67]. In addition, Relevant literature indicates that the VP1 monomer can form a G−H loop and have the ability to induce cellular and human immune responses [68,69]. The VP1 monomer is exposed to the surface by binding to the N−terminal of i301, while adding linkers to ensure its flexibility and full display of antigen epitopes [28]. In addition, adding a biological adjuvant to vaccines can enhance immunogenicity and achieve the best performance of target antigens [70]. nFLiC genetically fused to the C−terminal of i301 (VP1−i301−nFLiC) to enhance the immune effect of nanovaccine. The results showed that the VP1−i301−nFLiC fusion protein could also self−assemble into uniform NPs in vitro. Moreover, both groups of NPs showed strong thermal and storage stability, which is consistent with the previous reports of EBV and CSFV NPs designed based on i301 [66,71]. This finding also indicates that i301 has a high capacity to break through the size limit of heterologous targets and maintain strong stability, which may address the problem of cold−chain transportation, making it possible to develop more nanovaccines.

Further results revealed that NPs, not monomeric VP1, could significantly enhance the adsorption and phagocytosis of cells and promote the activation of APCs, including upregulating the expression level of MHC, surface markers (CD80 and CD86), and cytokines, which could significantly enhance antigen cross−presentation and protective immunity [72,73]. Notably VP1−i301 nFLiC NPs have a stronger stimulating effect compared with VP1−i301 NPs, indicating that nFLiC effectively stimulates TLR5 signal transduction and further enhances immune protection.

As expected, NPs have shown significant advantages in cellular and humoral immunity, because the designed NPs at 25–50 nm are optimal to access the lymphatic system where the majority of adaptive immunity initiates [29]. High levels of cytokine expression promote the proliferation and differentiation of T cells and induce Th immune response. A high titer of neutralizing antibodies plays a key role in resisting FMDV challenge, and the protection rate of VP1−i301−nFLiC NPs reaches 83%, which is the same as that of the inactivated FMDV group. nFLiC protein collaborates with NPs to exhibit excellent immune−response capabilities in vivo. The immune response of NPs has been significantly enhanced compared with monomeric antigens, which is consistent with previous reports of HBc VLP and SARS-CoV-2 NPs [74,75]. NPs can not only increase the titer or density of antigens, but also covalently connect biological immune enhancers to promote the induction of protective immune response [66]. These results provided sufficient evidence to prove that the subunit vaccine based on the NP strategy can simulate the natural conformation of pathogens, significantly improving the ability of antigen uptake, processing, and presentation, as well as inducing a high level of protective immune response in vivo [11,76].

However, the immune−response intensity generated by the NP group remains slightly lower than the inactivated vaccine. The immune response induced by the fusion protein containing only VP1 is relatively monotonous, whereas inactivated vaccines can effectively promote the rapid and effective activation of innate and adaptive immune responses. However, NP vaccines not only ensure immune efficacy while reducing immune costs, but also play the role of the DIVA vaccine. This finding prompts us to design and modify NP vaccines to induce stronger immune responses.

## 5. Conclusions

This study shows that the gene−encoded NPs display an antigen that is used for vaccine design and development, thereby forming nanovaccines with FMDV VP1 antigen and nFLiC. Compared with monomeric antigens, NPs have good thermal stability, and they enhance the uptake efficiency of cells, upregulate the transcription level of cytokines in vitro, and improve the antibody level in animals. Notably, VP1−i301−nFLIC was superior to VP1−i301 NPs. Moreover, in the animal model against FMDV challenge, the VP1−i301−nFLIC nanovaccine achieved comparable protective effects to inactivated FMDV, and the protection of VP1−i301−nFLIC NP group was better than that of VP1−i301 NP group. These results provide valuable guidance for the research and development of novel nanovaccines.

## Figures and Tables

**Table 1:** Primer sequences of BMDCs for qPCR.

Gene	Sequence
β−actin F	GCTGTCCCTGTATGCCTCT
β−actin R	TTGATGTCACGCACGATTT
MHC II F:	CTGTCTGGATGCTTCCTGAGTTT
MHC II R:	TCAGCTATGTTTTGCAGTCCACC
CD80 F	CCCCAGAAGACCCTCCTGATAG
CD80 R	CGAAGGTAAGGCTGTTGTTTG
CD86 F	GCCGTGCCCATTTACAAAGGCTCAA
CD86 R	TGTTACATTCTGAGCCAGTTTTATT
TNF−α F	GGCAGGTCTACTTTGGAGTCATTGC
TNF−α R	ACATTCGAGGCTCCAGTGAATTCGG
IL−6 F	TCCAGTTGCCTTCTTGGGAC
IL−6 R	GTGTAATTAAGCCTCCGACTTG
IFN−γ F	TCAGCTGATCCTTTGGACCC
IFN−γ R	CTCAGAGCTAGGCCGCAGG

**Table 2:** Primer sequences of guinea pigs for qPCR.

Gene	Sequence
GAPDH F	TGCCCCTATGTTCGTGATGG
GAPDH R	TGTGGTCATGAGTCCCTCCA
TNF−α F	CGCTCACACTCAGATCAGCTT
TNF−α R	GACGGTATGGGTGAGAAGCA
IFN−γ F	AATGACGAGCATGTCCAGCG
IFN−γ R	CTCTCCGGCTCTGAAACAGC
IL−12p 70 F	GTCACAAAGGAGGCGAGGTT
IL−12p 70 R	AGCAGGTGAAACGTCCAGAA

**Table 3:** Protection of guinea pigs after FMDV challenge.

Guinea Pigs	PBS	Inactivated FMDV	VP1	VP1−i301	VP1−i301−nFLiC
1	None	Partial	None	Full	Full
2	None	Full	Full	Full	Full
3	None	Full	None	None	Full
4	None	Full	Partial	Full	Partial
5	None	Full	Partial	Partial	Full
6	None	Full	Full	Full	Full
Rate of protection (%)	0	83.3	33.3	66.7	66.7

Note. Full: Neither the inoculated limbs nor the uninoculated limb have swollen and vesicles were judged as full protection. Partial: The inoculated limbs were inflamed and blistered, but other parts were not affected, and were judged as partial protection. None: Both the inoculated limbs and the uninoculated parts were infected, and were judged as unprotected.

## Data Availability

The data presented in this study are available on request from the corresponding author. The data are not publicly available due to privacy.
